# Efficacy of Acacia Gum Biopolymer in Strength Improvement of Silty and Clay Soils under Varying Curing Conditions

**DOI:** 10.3390/polym16192831

**Published:** 2024-10-07

**Authors:** Muralidaran Vishweshwaran, Evangelin Ramani Sujatha, Ateekh Ur Rehman, Arif Ali Baig Moghal

**Affiliations:** 1 Centre for Advanced Research in Environment, School of Civil Engineering, SASTRA Deemed University, Thanjavur 613401, India; 2 Department of Industrial Engineering, College of Engineering, King Saud University, Riyadh 11421, Saudi Arabia; 3 Department of Civil Engineering, National Institute of Technology Warangal, Warangal 506004, India; baig@nitw.ac.in

**Keywords:** polysaccharides, geotechnical, California bearing ratio, thermal curing, sustainable construction materials

## Abstract

Acacia gum (AG), a polysaccharide biopolymer, has been adopted to improve the strength of three cohesive soils by subjecting them to diverse environmental aging conditions. Being a polysaccharide and a potentially sustainable construction material, the AG yielded flexible film-like threads after 48 h upon hydration, and its pH value of 4.9 varied marginally with the aging of the stabilized soils. The soil samples for the geotechnical evaluation were subjected to wet mixing and were tested under their Optimum Moisture Content (OMC), as determined by the light compaction method. The addition of AG modified the consistency indices of the soils due to the presence of hydroxyl groups in AG, which also led to a rise in OMC and reduction in Maximum Dry Unit weight (MDU). The Unconfined Compressive Strength (UCS) and California Bearing Ratio (CBR) were determined under thermal curing at 333 K as well as on the same day of sample preparation. The least performing condition of the soil’s CBR was evaluated under submerged conditions after allowing the AG-stabilized specimens to air-cure for a period of 1 week. The UCS specimens tested after 7 days were subjected to the initial 7 days of thermal curing at 333 K. A dosage of 1.5% of AG yielded the UCS of 2530 kN/m^2^ and CBR of 98.3%, respectively, for the low compressible clay (LCC) after subjecting the sample to 333 K temperature for 1 week. The viscosity of the AG was found to be 214.7 cP at 2% dosage. Scanning Electron Microscopy (SEM), Fourier Transform Infrared Spectroscopy (FTIR), and average particle size determination revealed the filling of pores by AG gel solution, adsorption, and hydrogen bonding, which led to improvements in macroproperties.

## 1. Introduction

The development of a nation is greatly influenced by the presence of a well-established highway infrastructure, which ensures convenient connectivity and accessibility. The performance of a pavement relies heavily on the subgrade’s capacity to handle traffic loads. Subgrade failure is a significant cause of pavement deformation. The cost of constructing pavements can be significantly higher when dealing with soils that have poor engineering properties. Clays with subpar engineering properties pose a challenge in terms of supporting traffic loads, as their subgrade strength is insufficient. The strength and serviceability of the pavement over a design period are dependent on the structural condition of the subgrade soil, which is crucial for the highway infrastructure. Constructing efficient and long-lasting pavements on problematic fine-grained soil poses a significant engineering challenge. The cost of reconstructing a failed pavement due to deeper rutting deformation is INR 3000/m^2^ [1]. Efficient compaction techniques may not always result in the desired increase in soil strength for flexible pavement construction, even when applied to fine-grained soils. One of the main challenges faced when dealing with subpar geomaterials is the increase in material costs, along with the associated pollution and the lack of nearby soils with desirable engineering properties. Soils that have naturally poor engineering properties do not necessarily need costly deep foundations or soil replacement [2]. Enhancing soil properties is essential for achieving the desired geotechnical engineering properties for pavement applications.

It is important to ensure that sustainable construction practices prioritize the protection of groundwater and soil without compromising energy and resource savings [3]. Using biological methods is a sustainable approach to enhancing soil parameters. Biopolymers are naturally occurring polymers made up of repeated monomeric units found in plants, animals, and microorganisms [4]. They have the capacity to create interconnected networks and gels as a result of their molecular structure. They have the ability to create hydrogels and have a high molecular weight. Due to their unique biological and functional characteristics, biopolymers have found extensive applications in food, packaging, manufacturing, and paper, and have proven to be a viable alternative to synthetic polymers [5].

The utilization of Rhizobium tropici biopolymer in slope treatment, combined with hydroseeding, has proven to be highly effective in promoting vegetation growth, reducing surface roughness, minimizing soil loss, and improving slope stability for a period of more than 3 years [6]. The application of alginate biopolymer on silty loam soil bricks resulted in a significant reduction in linear shrinkage, from 9% to 5%. Additionally, it led to notable improvements in both compressive and flexural strength, with their values increasing by 92.9% and 196.9%, respectively [7]. The incorporation of guar gum biopolymer along with lime led to a significant enhancement in the soil’s UCS and CBR by a remarkable 650% [8]. Molfetta and Sethi [9] utilized guar gum in their study on clamshell excavation for the construction of a permeable reactive barrier. This innovative approach successfully remedied the contamination of an aquifer. By using succinoglycan biopolymer in the treatment of silty soils, Ringelberg et al. [10] were able to increase the peak stress and strain energy of the silty soil by a factor of four. A substantial increase in the elastic modulus of modified starch-stabilized silty soil was seen, with a fourfold increase observed over the unstabilized conditions [11]. Kwon et al. [12] enhanced the shear strength of an onshore clay by 3900% by the inclusion of xanthan gum biopolymer. The choice of enzyme-induced carbonate precipitation led to improvements in crust thickness (142%), penetration resistance (800%), and calcium carbonate content (1.4%), which promoted the better erosion resistance of the coarse-grained soil [13]. Maaitah [14] stabilized an HCC soil by combining sodium silicate with lime and improved the CBR of the soil by 153%.

AG is sustainably produced as it is taken from trees without causing harm to the environment. Since the gum trees continue to grow even after they have been tapped, they can be considered a renewable resource [15]. AG is an indigenous, biodegradable polymer that is environmentally benign has no negative impact on the soil ecosystem and is a favorable option for environmentally friendly soil improvement techniques. This biopolymer is produced by the sap of Acacia plants, particularly Acacia senegal and seyal, and has historically been used for many years in medicine and cosmetics [16]. The versatility of AG is shown in its application across multiple domains, and it can serve as an adhesive, thickener, stabilizer, and emulsifier. Finalizing a soil binder for field utilization requires the compatibility assessment of the binder with the weak soil in terms of soil-binder interactions, ease of handling, and mixing for the intended application. There is a demand for a soil stabilizer that is sustainable and environmentally friendly. AG produces soft, flexible gels upon hydration and easily dissolves, which are more suited to the versatility of soil texture and composition [17,18]. Achieving uniform workability with a highly viscous xanthan gum is a challenge, and unlike non-ionic biopolymers, the anionic AG has the potential to form effective electrostatic bonds with positively charged clay minerals [19]. The AG is also stable across varying temperatures and pH compared to most biopolymers and is a potential binder for soil strengthening [20]. The AG can also be procured locally due to the abundance of AG trees, which requires no chemicals and uses minimum electricity for production, thus leaving a very low carbon emission [21]. Oluremi and Ishola [22] have improved the CBR of a lead-contaminated clay under soaked and unsoaked conditions by varying the compaction energies, while the wind erosion resistance of AG-stabilized sand was demonstrated by Dagliya and Satyam [23] as well as by Lemboye et al. [24]. The utilization of three different types of environmental aging conditions was adopted to assess the strength of the AG-stabilized soils: (i) unconfined room temperature aging followed by testing; (ii) aging by application of heat at 333 K followed by testing; and (iii) unconfined room temperature aging before submergence followed by testing. The unconfined room temperature aging before testing permits the analysis of AG-stabilized soils’ resistance to compressive failure depending on the curing duration. Application of heat at 333 K could unravel the possibility of accelerated strength development and the extent of effectiveness of polymerization and stabilization at differing curing durations. Submergence of the specimens was performed after 1 week of air curing in order to simulate the worst scenario of soil’s resistance against the compressive loads. The short-term objective of the work aims to assess the strength of AG-stabilized soils under these three diverse environmental aging conditions, which include the associated mechanisms in the initial and intermittent days, whereas the future scope of the work involves the field application of AG-stabilization for strength improvement, freeze–thaw durability analysis, analysis of strength loss after wet–dry cycles, and swell–shrink and consolidation parameters’ assessments. In this study, AG, a polysaccharide biopolymer, has been utilized to improve the specific geotechnical characteristics of three fine-grained soils under different environmental aging conditions.

## 2. Materials

Three different cohesive soils have been chosen for the purpose of soil enhancement and for understanding the soil–AG interaction under a variety of aging periods with differing temperature environments. The AG biopolymer consists of β-D-galactopyranosyl units as its main structure, with additional side chains composed of arabinose, galactose, and rhamnose [25]. The high molecular weight and low viscous gel of AG are attributed to its complex polysaccharides, primarily arabinogalactan-proteins, when hydrated. The biramous shape enables a multitude of interactions with molecules, including soil particles. AG has a high water absorption capacity, allowing it to swell and create a gel-like substance that can cover the gaps between soil particles [26]. The elemental composition of AG and the soils is presented in Table 1 below.

## 3. Methodology

**Figure 1:**
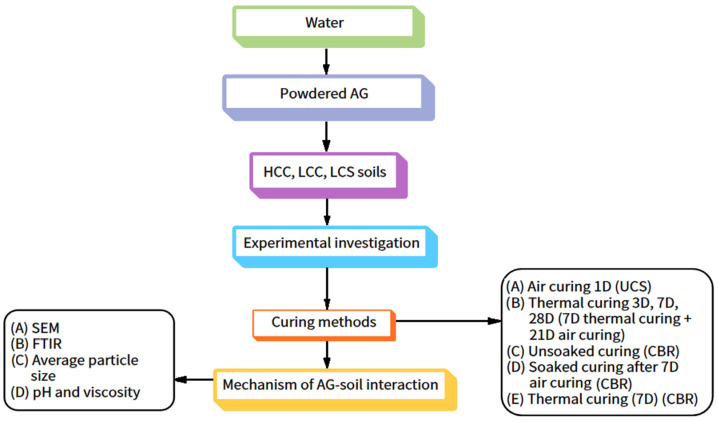
Workflow methodology.

The workflow of the AG stabilization of the three soils is shown below in Figure 1.

The treatment of the soil specimens was accomplished by the use of the wet mixing method, by which water was carefully incorporated into the powdered biopolymer at its OMC, reserving a 1% reduction in OMC for later soil mixing. The soil mixture was sealed for 2 h to ensure no moisture was lost and compacted to OMC for various dosages [27]. OMC and MDU were determined based on the light compaction technique by application of rammer blows to the collected soil. The liquid limit was determined from the Casagrande liquid limit apparatus, and the plastic limit was determined from the breaking of the soil threads at 3 mm diameter. The UCS was determined after 120 min of sample preparation. A heat-based treatment condition at 333 K was adopted for initial aging of 3 and 7 days [28]. The 28-day specimen was thermal-cured for 7 days and air-cured for the next 21 days for the determination of UCS. The soaked CBR of the soil was determined by air-curing the samples for 7 days and submerging them for 4 days before testing. CBR testing was also performed without delay after the sample was prepared [29]. Thermal curing of 7 days for CBR specimens was also conducted. Different biopolymer dosages and curing periods were adopted to determine the optimal performance of the AG-stabilized soils. The requirement of water for the soil specimens varies depending on the dosages of AG, and thus the OMC has been determined for all the dosages. A total of 60 UCS specimens and 45 CBR specimens were tested, and the mean value of the three specimens was adopted for each dosage. The standard deviation of the AG-stabilized HCC, LCC, and LCS soils is 5%. The soaked CBR is conducted to interpret the engineering behavior of the soil subgrade in the event of soil submergence, i.e., its worst condition [30]. The Atterberg’s limits, OMC and MDU, UCS and CBR, were evaluated by the guidelines outlined in Bureau of Indian Standards IS 2720: Parts 5, 7, 10, and 16 [31,32,33,34] respectively. FTIR, SEM, and average particle size determination were performed with Perkin Elmer, Tescan Oxford, and Malvern Panaltical, respectively [35]. pH and dynamic viscosity of the AG were determined by a pH meter and Brookfield viscometer, respectively [36].

## 4. Results and Discussion

### 4.1. Plasticity Characteristics

**Figure 2:**
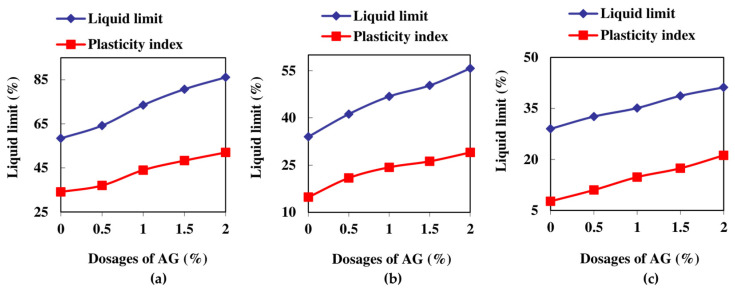
Consistency limits of the AG-stabilized soils: (**a**) HCC; (**b**) LCC; (**c**) LCS.

Although AG solutions did not form highly viscous gels compared to other gum-based polysaccharides such as xanthan gum and guar gum, the stabilized soils displayed a thick, rigid texture during the mixing stage of liquid and plastic limit determination [37]. The maximum liquid limit observed for the soils was determined to be 86.1%, 55.7%, and 41.2%, respectively, for the soils ranging from high plastic soils to low plastic soils. Additionally, their plasticity index values were measured to be 52%, 29%, and 21.2%, respectively. The maximum increase in LL and PI was observed to be 63.8% and 175.3%, respectively, for the LCC and LCS soils at the 2% dosage. A steady increase with the higher dosages was observed for all three soils stabilized with AG, with the maximum value at an AG dosage of 2%. The AG exhibits a high degree of hydrophilicity as a result of the many hydroxyl groups inside its molecular structure. The increasing dosages of AG had a contiguous impact on the consistency attributes of the soils, resulting in the clays needing more water [38]. Figure 2 shows the results of the liquid and plastic limits of the AG-stabilized fine-grained soils.

### 4.2. Compaction Characteristics

**Figure 3:**
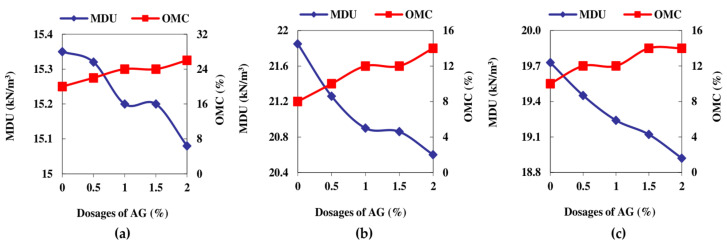
OMC and MDU of the AG-stabilized soils: (**a**) HCC; (**b**) LCC; (**c**) LCS.

The MDU of LCC, LCS, and HCC soils were found to be 21.85 kN/m^3^, 19.73 kN/m^3^, and 15.35 kN/m^3^, and their corresponding OMCs were determined to be 8%, 10%, and 20%, respectively. The AG-stabilized soils showed a gradual increase in OMC with increasing AG dosages. In addition to the hydrophilicity, the viscosity of the AG also contributed to the increase in OMC. The AG solution also has a tendency to cause the soil particles to separate when there is more water present [39]. A slight decrease in MDU was observed for the modified soils as the concentrations increased, which can be attributed to the following factors: The hydrated products enhance the binding of the AG-stabilized soils, resulting in increased stability of the clay–polymer complex, but the MDU decreases. AG with its low specific gravity of 1.4 could have reduced the MDU of its stabilized soils. The AG–soil workability is affected by increasing dosages, which also reduces the MDU, and this reduction in MDU is presented in Figure 3 below.

### 4.3. UCS

#### 4.3.1. Strength Characteristics

**Figure 4:**
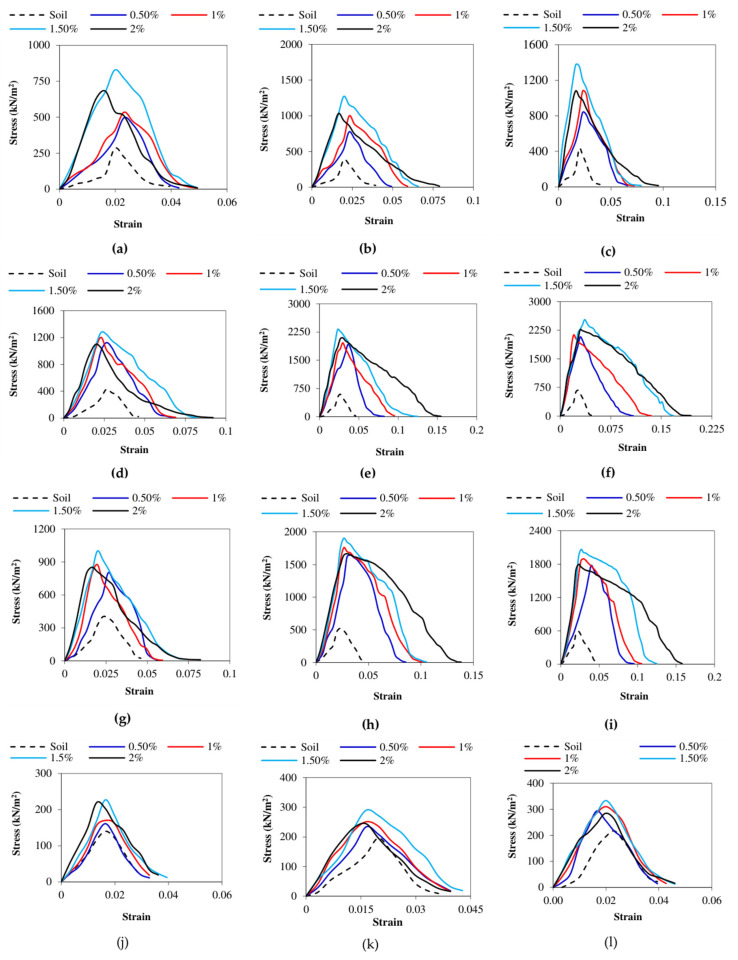
UCS curves of AG-stabilized soils: (**a**) HCC—3D at 333 K; (**b**) HCC—7D at 333 K; (**c**) HCC—28D at 333 K; (**d**) LCC—3D at 333 K; (**e**) LCC—7D at 333 K; (**f**) LCC—28D at 333 K; (**g**) LCS—3D at 333 K; (**h**) LCS—7D at 333 K; (**i**) LCS—28D at 333 K; (**j**) HCC—1D; (**k**) LCC—1D; (**l**) LCS—1D.

The LCC, LCS, and HCC soils that were tested on the same day after the cylindrical specimens’ preparation and 120 min of air curing revealed values of 316 kN/m^2^, 268 kN/m^2^, and 189 kN/m^2^, respectively. The application of heat to facilitate curing at 333 K yielded the highest UCS response among all the curing conditions. The rise in strength of LCC was particularly well observed for the heat treatment at 333 K for 1 week followed by the exposure of the LCC samples to atmospheric air for three subsequent weeks, leading to a strength of 2530 kN/m^2^ with a 1.5% addition of AG. This strength enhancement was in contrast to the LCC as well as HCC and LCS samples, which were evaluated for strength after 120 min post molding. The LCC, HCC, and LCS showed greater efficacy in resisting the compression for the first seven days compared to the subsequent days, even though the shearing resistance was greater than the control samples without aging. The UCS increase occurs as a result of the interparticle interaction between AG and the soil along with the decrease in moisture content in the stabilized specimens. The UCS curves are shown in Figure 4 below.

**Figure 5:**
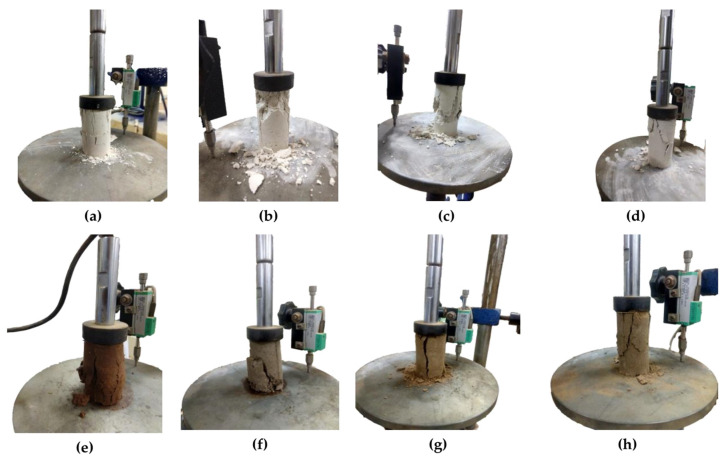
UCS curves of AG-stabilized soils: (**a**) HCC—3D at 333 K; (**b**) HCC—7D at 333 K; (**c**) HCC—28D at 333 K; (**d**) HCC—1D; (**e**) LCC—1D; (**f**) LCS—1D; (**g**) LCC—28D at 333 K; (**h**) LCS—28D at 333 K.

Figure 5 reveals the improvement in the toughness of the AG-stabilized soils with the increase in the curing time. The deformation modulus (E_50_) of the AG-stabilized HCC, LCC, and LCS soils increased by 718%, 371%, and 206%, respectively after 28 days of strength assessment. The failure of the specimens in HCC soil reveals brittle failure for the heat-cured specimens, whereas the specimen tested after 120 min exhibited shear failure. LCC and LCS soils failed in shear predominantly in all the curing days and conditions. The failure of the UCS specimens is shown in Figure 5 below.

#### 4.3.2. Mechanism of Strength Improvement

After applying an AG solution to the soils, the dissolved particles infiltrate the pores and saturate the surface. The electrical charges of the anionic biopolymer, the inherent cations in clay, and the charge density on clay sheet surfaces increase the reactivity of fine-grained soils [40]. AG also forms a surface layer around soil particles, linking them and allowing direct chemical reactions to agglomerate them [22]. Air-drying may form a solid layer on a sample’s outside, preventing inner curing. Both the external and interior cementitious processes occur consistently when the sample is subjected to heat treatment at 333 K [38]. Moreover, AG serves as a surface layer that envelops soil particles, forming linkages and enabling direct chemical interactions to agglomerate dispersed particles [22]. HCC with a pH of 6.1 could ionize carboxyl groups (–COOH) in AG, generating carboxylate ions (–COO^−^) that interact with clay cations. LCC with a pH of 6.8 could facilitate ionic crosslinking between AG and clay particles due to their pH differences, which increase electrostatic interaction. Silty soils have low charge density and cation exchange capacity; therefore, physical encapsulation and coating are more prominent than the electrostatic bonds [41,42]. Cheng and Geng [43] utilized a gellan gum biopolymer for the stabilization of an HCC and recorded a UCS of 1020 kN/m^2^ after 28 days of curing. This increase in UCS from 410 kN/m^2^ was attributed to the hydrogen bonding between the HCC and the gellan gum. Azimi et al. [44] utilized chitosan biopolymer to enhance the UCS of clay by 106% and the strengthening mechanism was attributed to electrostatic attraction and flocculation of the clay–polymer complex. Armistead et al. [45] cited covalent and electrostatic bio–mineral interactions for the improvement of UCS from 0 kN/m^2^ to 3700 kN/m^2^ upon stabilization using locust bean gum. Liu et al. [46] emphasized the integrated LCC-locust bean gum matrix in achieving enhanced UCS, tensile strength and shear strength parameters due to the clustering of the stabilized LCC by interparticle interaction. Similar mechanisms were observed for AG’s strengthening of the three soils and are described in the subsequent sections.

#### 4.3.3. Effect of Aging and Temperature

Stabilized soil strength depends on the underlying soil’s geotechnical qualities. Allowing time for the hydrogel to dehydrate is important since moisture content affects curing time [40]. The AG–soil matrix becomes stronger as the curing days progress, thanks to the drying process that reinforces the fibrous linkages between the soils and the AG. The AG has the ability to fill voids and bind to the fine-grained soils, resulting in a solid and cohesive structure over time [47]. One possible reason for this could be the formation of hydrated bonds and agglomeration caused by the introduction of biopolymers into fine-grained soils. In a recent study, Reddy et al. [48] found that the biopolymer films remain intact over an extended period of time, even after being exposed to soil, due to the transformation and stiffness from the curing process of the specimens. Feng et al. [49] performed UCS of a xanthan gum-stabilized soil under varying temperatures from 20 °C to 60 °C and noted that the 60 °C offered the optimum result. The importance of the removal of residual moisture content, which contributed to the maximum strength increase of 3.6 MPa, was elucidated by Feng et al. [49]. Being an anionic biopolymer such as AG, elevating the temperature to 333 K causes conformational shifts in xanthan molecules, which in turn strengthens the bonding and interactions. The lubricating action of Persian gum beyond the optimum dosage was reported by Fatehi et al. [50], which led to the separation of soil particles beyond the optimum dosage. This variation in the workability of the gum-based stabilization promoted lubrication instead of flocculation when the AG dosage exceeded 1.5%. AG diminishes clay soils’ specific surface by filling holes, changing their physical characteristics [51]. Latifi et al. [47] adopted a similar anionic biopolymer (xanthan gum) for HCC soil and reduced the specific surface by particle diameter variation with curing day, hence altering the soil’s physical properties. Applying heat increased compressive strength in the specimens, which Chang and Cho [28] attributed to water removal making them more rigid. Elevating the temperature of the processed soil leads to a reduction in the remaining moisture content, thereby enhancing its strength [52]. This leads to a coating of the soil surfaces and the entanglement of soil grains with AG, resulting in enhanced rigidity over time [48]. The gel threads of AG enhance the soil voids and the surroundings, resulting in an improved strength of the AG-stabilized soils. There is no change in the stability of the AG–soil fibrous connections after seven days of air curing and twenty-one days of air curing. The aggregation of the AG is a result of its mobility and the interaction between the molecules, resulting in the formation of stable confluent areas [53].

### 4.4. CBR

**Figure 6:**
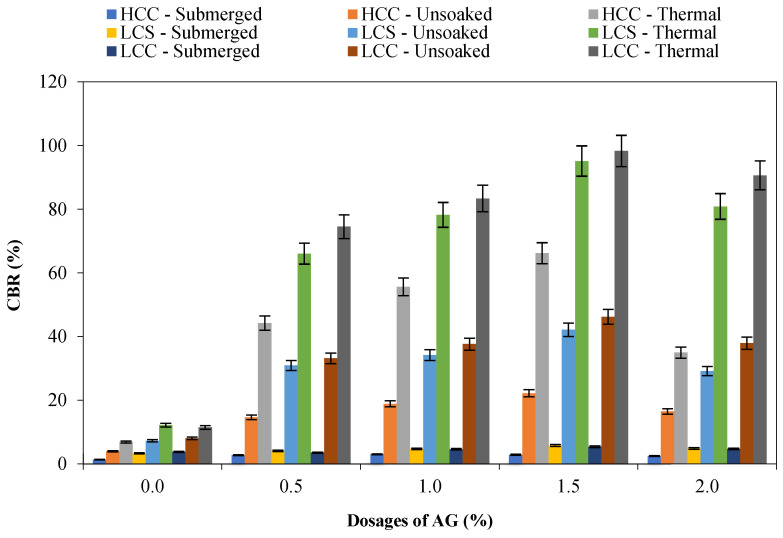
CBR of AG-stabilized soils.

The soaked CBR reached the mandatory requirement of 5% as per the Indian Roads Congress [54] for 1.5% dosage for the LCS and LCC, showing an improvement of 72.4% and 43.77%, respectively. However, the AG stabilization resulted in an increase in the CBR by 625.47% and 753% on LCS and LCC soils under dry test conditions. The application of heat treatment at 333 K has further enhanced the CBR for all the soil types tested. The exceptional increase in the CBR of the stabilized soils can be attributed to the thermosetting process caused by heat absorption. Increasing the temperature of the stabilized soil leads to a reduction in residual moisture content, resulting in a boost in strength. Khaleghi and Heidarvand [55] adopted a non-ionic Salep biopolymer and increased the unsoaked CBR from 25.3% to 300% and the soaked CBR from 19.5% to 75%, respectively. The increase in CBR was attributed to the electrostatic forces and formation of bridges in the stabilized soil. Hamza et al. [56] noted a rise in unsoaked and soaked CBR by 300% and 200%, respectively, upon stabilizing an HCC with guar gum. The findings revealed that a reduction in the number of voids in the soil and filling them creates robust connections within the guar gum-stabilized HCC. Sawwaf et al. [57] stabilized an LCC soil with gelatin and sodium alginate and improved the CBR by more than 100% for both biopolymers. The increased results of the CBR were attributed to the electrostatic, hydrogen bonding, and van der Waals forces between the stabilized soil and the biopolymer. These strands then intertwine with the soil particles and effectively fill the gaps between them. Both the control and AG-stabilized specimens underwent a significant reduction in CBR post-submergence. The AG–soil matrix hydrates and is weakened by the reduction in cohesion and electrostatic attraction. Figure 6 shows the results of CBR of the AG-stabilized soils.

### 4.5. pH and Viscosity

The pH of the HCC was 6.1, the LCC was 6.8, and the LCS was 7.05, while the AG was at a much lower pH of 4.95. Following the air curing period of 28 days, there was minimal alteration in the pH level of the soil. Table 2 illustrates the pH fluctuations of the AG during the period of the initial day and after 28 days.

**Figure 7:**
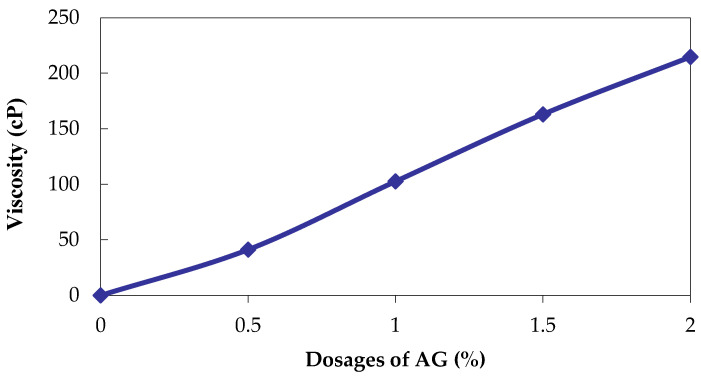
Viscosity of AG.

The results indicate that the addition of AG does not adversely affect the pH of the soil. The pH level significantly influences the charge and stability of the kaolin–AG combination, and the interaction between clays and negatively charged AG is contingent upon the concentration of hydrogen ions present in the system [41]. The charge of the AG is influenced by its functional group, which in turn is influenced by its pH. Research has demonstrated that kaolin has the ability to absorb amino acids across a wide range of pH levels [58]. The absorption of amino acids in significant quantities using kaolin is due to its external positive charge [59]. The effectiveness of the process is highest within the pH range of 3 to 7 due to the migration of ions. At the boundaries of clay, AG, with its low pH, adheres to them due to the presence of hydrogen bonds [60]. The bonding between the silicate layers of clay is facilitated by van der Waals forces, resulting in increased absorption energy [61]. The AG biopolymer solution displayed little changes in viscosity within the concentration range of 0.5% to 2%. The highest concentration of AG resulted in a maximum viscosity of 214.7 cP. Viscosity increases the UCS by facilitating the AG to bond with the geomaterials. Vishweshwaran and Sujatha [62] highlighted the synergistic effects of viscosity and molecular weight on HCC soil’s shear resistance due to the increase in thread linkages of a protein biopolymer in HCC soil which enhanced the soil’s ability to resist shear forces. Figure 7 shows the results of the viscosity of AG for different dosages.

### 4.6. FTIR

**Figure 8:**
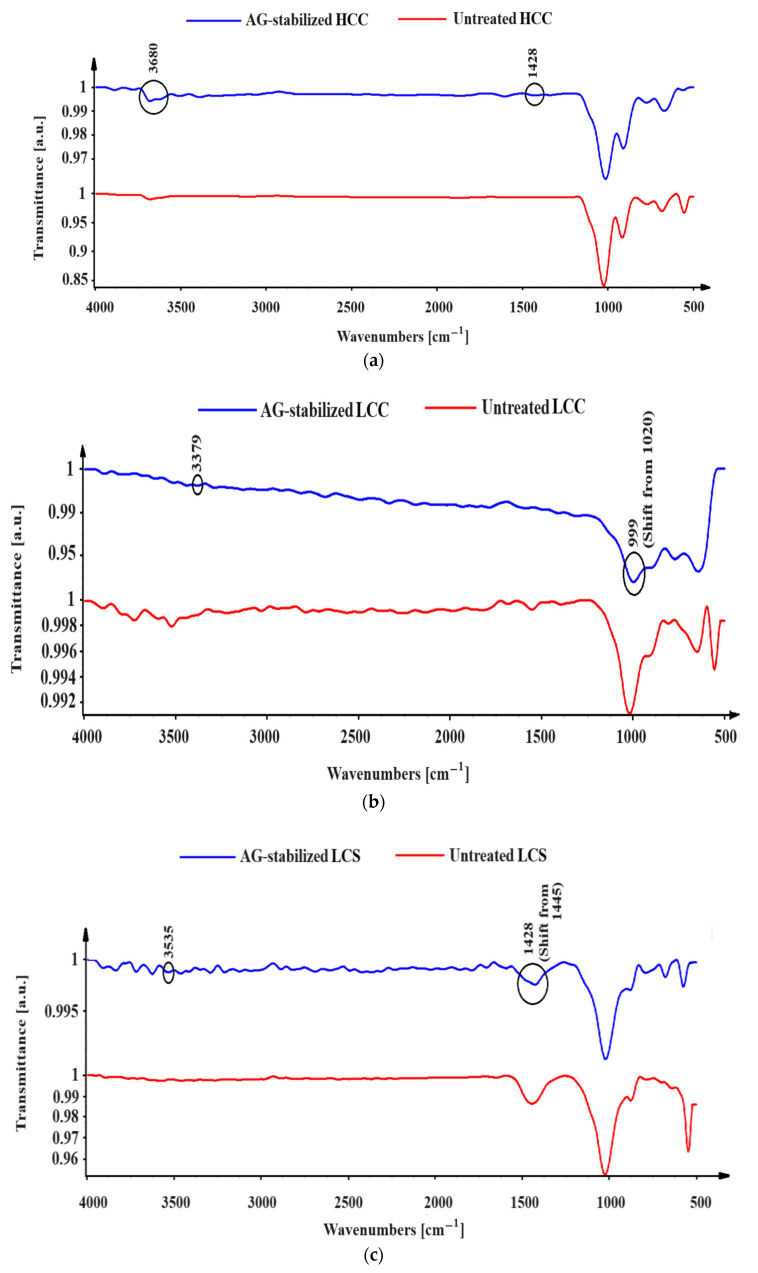
FTIR spectra of unstabilized and AG-stabilized soils: (**a**) HCC; (**b**) LCC; (**c**) LCS.

The wavenumbers corresponding to the molecular vibrations of the clay–biopolymer interaction are depicted in Figure 8. The addition of AG to HCC soil leads to the formation of FTIR peaks at 3680.18 cm^−1^ and at 3379 cm^−1^ for LCC soil, which signifies the stretching vibration of Si-OH bonds, contributing to hydrogen bonding [63]. The hydroxyl (-OH) and carboxylate (-COO^−^) groups present in AG reinforce the soil pores. The addition of AG to LCC results in the interaction between the carboxylate ions (-COO^−^) of the gum and the positively charged sites on the clay particles due to the shift in wavenumber from 1445 cm^−1^ to 1428 cm^−1^ [63]. Electrostatic attraction occurs when negatively charged carboxylate ions interact with positively charged sites on clays [64]. The change in wavenumber from 1020 cm^−1^ to 999 cm^−1^ for the LCS soil reveals a stretching mode of vibration of the C-O group, which has the ability to establish hydrogen bonds with silanol (Si-OH) or aluminol (Al-OH) groups on silty clay. The C-O-C and C-O groups of AG can adhere to silty clay surfaces through weaker forces and hydrogen bonding, denoted by the wavenumber at 999 cm^−1^ in the FTIR spectrum [63].

### 4.7. SEM and Average Particle Size

**Figure 9:**
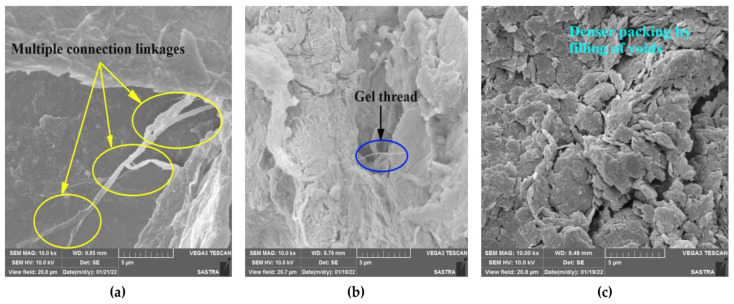
SEM of AG-stabilized soils. (**a**) HCC; (**b**) LCC; (**c**) LCS.

The unaggregated, dispersed clay soils containing empty spaces were not only filled but also clumped together after stabilization of the soils with AG. The AG filled the empty established connective linkages and hydrogen bonds with the fine-grained soils [47]. The agglomeration of the soil–AG complex is evident from the presence of various strands of threads passing through the soil structure, as shown in Figure 9.

**Figure 10:**
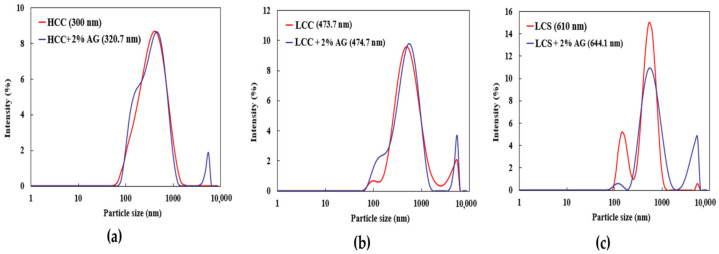
Zetasizer results of AG-stabilized soils: (**a**) HCC; (**b**) LCC; (**c**) LCS.

The results of the experiment show the changes in the structure that occurred after the AG treatment over a 28-day period. The gel threads of AG enhance the soil matrix, resulting in increased strength. The soil matrix becomes stronger as the curing days progress, and the fibrous linkages stiffen during the drying process. Furthermore, the AG biopolymer has the ability to adsorb onto the surface of clay soils, resulting in the formation of a solid and cohesive mass over time. The AG-stabilized HCC, LCC, and LCS showed an increase in particle sizes, as shown in Figure 10. This finding aligns with the SEM and FTIR analyses, which suggest a robust coating of the biopolymer onto the soils.

## 5. Conclusions

The mainstream adoption of biopolymers in geotechnical applications is not prevalent because of the following factors: The interaction of biopolymers with soil, especially fine-grained clays, can lead to increased viscosity, resulting in uneven mixing and skewing the evaluation of potentially useful polymers. The lack of standardized protocols for forecasting the efficacy of different biopolymer stabilizers, and the curing of biopolymer-stabilized soils are other critical aspects that require consideration. The long-term freeze–thaw resistance of the AG-stabilized soils and its engineering behavior under compressive stresses are to be evaluated. The in situ utilization of the AG stabilization requires comprehensive field assessments of the engineering performance, economy, and environment. The synergistic effects of AG with natural fibers, nanomaterials, and other environmentally friendly materials may contribute to new prospects in sustainable geotechnics. The dynamic behavior of the AG-stabilized soils under repetitive load applications for pavements and other sectors is yet to be analyzed in detail. Research collaborations with environmental engineering, materials science, and policy-making can help build the diverse knowledge base needed to develop geotechnical solutions for infrastructure issues. A thorough testing technique that integrates the distinct characteristics of diverse biopolymers and their engineering behavior with soils is necessary to assess their suitability for in situ application. The cost of biopolymers decreased tenfold from 2000 to 2007, which is attributable to the extensive utilization in pharmaceuticals, printing, textiles, and building sectors. High-purity, medicinal-grade biopolymers may be unnecessary for soil applications, and hence, tailored-grade manufacturing for specific applications could result in a decrease in prices. Interdisciplinary teamwork holds considerable potential for future geotechnical research, especially collaboration with the environmental sciences. Integrating geotechnical engineering with environmental research, materials science, and policy-making can help build the diverse knowledge base needed to develop geotechnical solutions for infrastructure issues.

The AG biopolymer has the ability to enhance challenging geomaterials under dry conditions favorably and offers average subgrade strength under soaked conditions depending on the soil properties. Studies on AG utilization in erosion control have been successfully performed by other researchers in this field. This work is an attempt to assess the suitability of the AG biopolymer under diverse curing modes and its corresponding strength determination. A mere quantity (1.5% dosage) is sufficient to achieve its optimum performance, beyond which workability challenges and marginal strength reduction are observed. Similar to other water-affine polysaccharides, the LL and OMC reached the maximum of 86.1% and 26%, respectively, for HCC soil. Marginal reductions in MDU and pH were noted for all dosages and for all the soil types, with the reduction in pH being negligible until 28 days. This finding, along with varying curing types, highlights the environmental applicability of the AG stabilizing binder. Within 2 h of the mixing of the AG, the UCS was improved by 62.1% for HCC, 53.5% for LCS, and 47.9% for LCC soils. Under all the mentioned CBR testing conditions and for all the soil types, the CBR was improved; the minimum, intermediate, and highest improvements were observed for soaked, dry, and thermal curing conditions, respectively. The highest CBR of 98.3% was noted for the LCC after heat application for a week followed by immediate testing.

## Figures and Tables

**Table 1:** The chemical makeup of the elements.

Soil	C (%)	O (%)	Ca (%)	Si (%)	Al (%)	Fe (%)	Na (%)	Mg (%)	Trace Elements
High compressible clay (HCC)	15.85	60.09	-	11.6	11.31	-	-	-	1.15
LCC	-	65.08	-	17.58	9.28	3.89	1.06	1.58	1.53
Low compressible silt (LCS)	14.82	54.69	13.5	6.93	3.11	2.64	-	1.78	2.53
AG	46.98	51.38	-	-	-	-	-	-	1.64

**Table 2:** pH of soils and AG-stabilized soils.

Soil Type	Soil		Soil + 0.5%AG		Soil + 1%AG		Soil + 1.5%AG		Soil + 2%AG	
	1D	28D	1D	28D	1D	28D	1D	28D	1D	28D
HCC	6.1	6.1	6.07	6.06	6.05	6.05	6.03	6.02	6.02	6.01
LCC	6.8	6.8	6.78	6.77	6.76	6.75	6.75	6.73	6.72	6.70
LCS	7.05	7.05	7.04	7.04	7.02	7.02	7.0	6.99	6.97	6.97

## Data Availability

All the relevant data are available as part of the manuscript.
